# Supplementary material for: Nod1-dependent NF-kB activation initiates hematopoietic stem cell specification in response to small Rho GTPases
Source: Nat Commun. 2023 Nov 23;14:7668. doi: 10.1038/s41467-023-43349-1 (PMC10667254; doi:10.1038/s41467-023-43349-1)
Supplement: Supplementary file 1 — Supplementary Information file [file 41467_2023_43349_MOESM1_ESM.pdf]

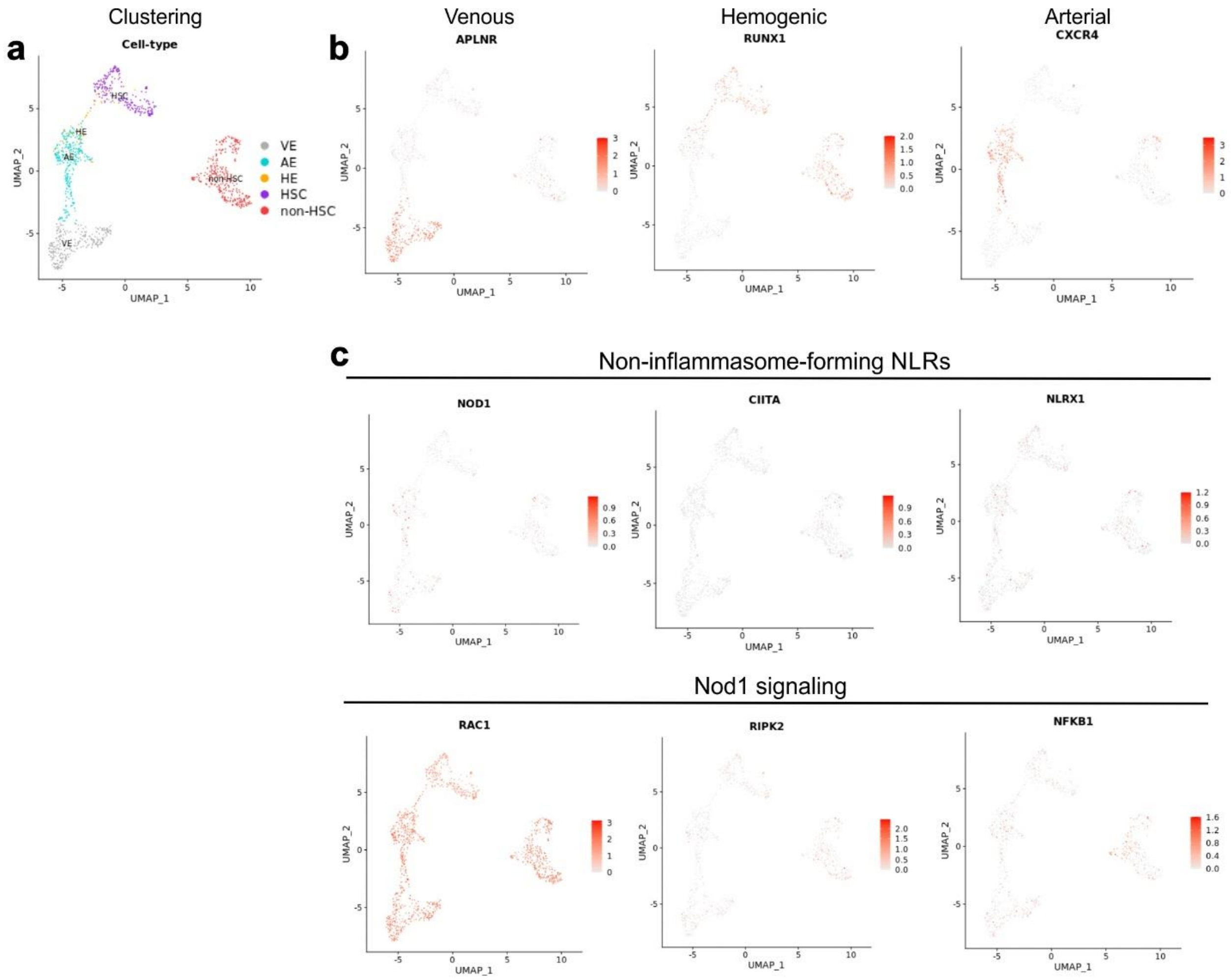

Supplementary Figure 1.
(a-c) UMAP visualization of the expression of indicated genes from human hemato-vascular populations from CS14–15 AGM tissues. Venous, hemogenic, and arterial markers are shown. Each dot represents one cell. In **b** and **c**, grey denotes minimal expression, orange intermediate, and red high. VE, venous endothelium; AE, arterial endothelium; HE, hemogenic endothelium; HSC, hematopoietic stem cells. Data extracted from https://singlecell.mcdb.ucla.edu/Human-HSC-Ontogeny/

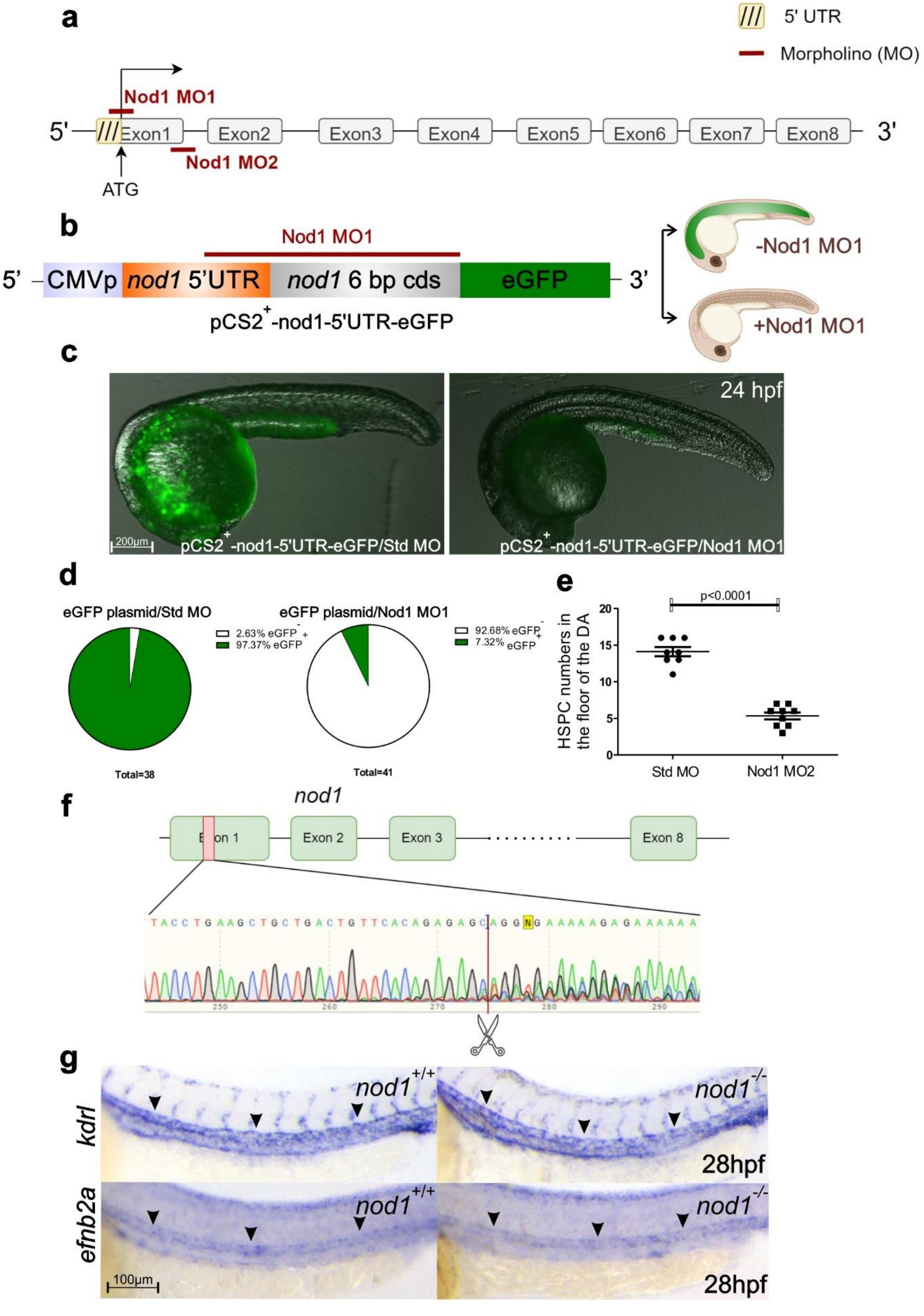
a
5' UTR
Morpholino (MO)
Nod1 MO1
5'
Exon1
Exon2
Exon3
Exon4
Exon5
Exon6
Exon7
Exon8
3'
Nod1 MO2
ATG
b
Nod1 MO1
5'
CMVp
nod1 5'UTR
nod1 6 bp cds
eGFP
3'
pCS2+-nod1-5'UTR-eGFP
-Nod1 MO1
+Nod1 MO1
c
24 hpf
200μm
pCS2+-nod1-5'UTR-eGFP/Std MO
pCS2+-nod1-5'UTR-eGFP/Nod1 MO1
d
eGFP plasmid/Std MO
2.63% eGFP-
97.37% eGFP+
Total=38
eGFP plasmid/Nod1 MO1
92.68% eGFP-
7.32% eGFP+
Total=41
e
p<0.0001
HSPC numbers in the floor of the DA
20
15
10
5
0
Std MO
Nod1 MO2
f
nod1
Exon 1
Exon 2
Exon 3
Exon 8
TACCTGAAGCTGCTGACTGTTCACAGAGAGCAGGNGAAAAAGAGAAAAAA
250
260
270
280
290
g
kdrl
nod1+/+
nod1-/-
28hpf
efnb2a
nod1+/+
nod1-/-
100μm
28hpf

Supplementary Figure 2.

(a) Schematic of binding of Nod1 MO1 (translation blocking morpholino) and Nod1 MO2 (splice blocking morpholino) to *nod1* immature mRNA. **(b)** Schematic representation of the validation strategy for Nod1 MO1. 324 bp upstream the *nod1* start codon were cloned into pCS2$^+$ under the CMV promoter (CMVp) following the first 6 nucleotides of the *nod1* coding sequence and driving eGFP expression (herein named pCS2$^+$-nod1-5'UTR-eGFP). One-cell state embryos were injected with this construct in the absence or presence of Nod1MO1. eGFP expression failed in the presence of Nod1MO. Illustrations created with BioRender.com. **(c)** Representative fluorescence images of 24 hpf embryos injected with pCS2$^+$-nod1-5'UTR-eGFP and co-injected with control Std MO or Nod1 MO1. n=40 per condition. **(d)** Percentage of eGFP$^+$ embryos quantified from (c). **(e)** Quantification of *cd41*$^+$*, kdrl*$^+$ HSPCs from 48hpf *cd41:eGFP; kdrl:mCherry* double-transgenic embryos injected with Std MO or Nod1 MO2 and imaged by live confocal microscopy. n=8, Std MO; n=9, Nod1 MO2. **(f)** Validation of *nod1 gRNA*. A *nod1* gRNA was designed to target exon 1 of the *nod1* gene. PCR amplicons from *nod1* exon1 targeted embryos were Sanger sequenced and the results revealed sequences containing indel mutation at predicted site. **(g)** *nod1*$^{+/+}$ and *nod1*$^{-/-}$ embryos examined by WISH for *kdrl* and *efnb2a* expression in the trunk at 28hpf. Arrowheads denote *kdrl*$^+$ or *efnb2a*$^+$ expression. n=15 per condition. All views are lateral, with anterior to the left. Images are representative of two independent experiments. All quantifications are represented with mean ± SEM. Data was analyzed by unpaired two-tailed T-test (e). Source data are provided as a Source Data file.

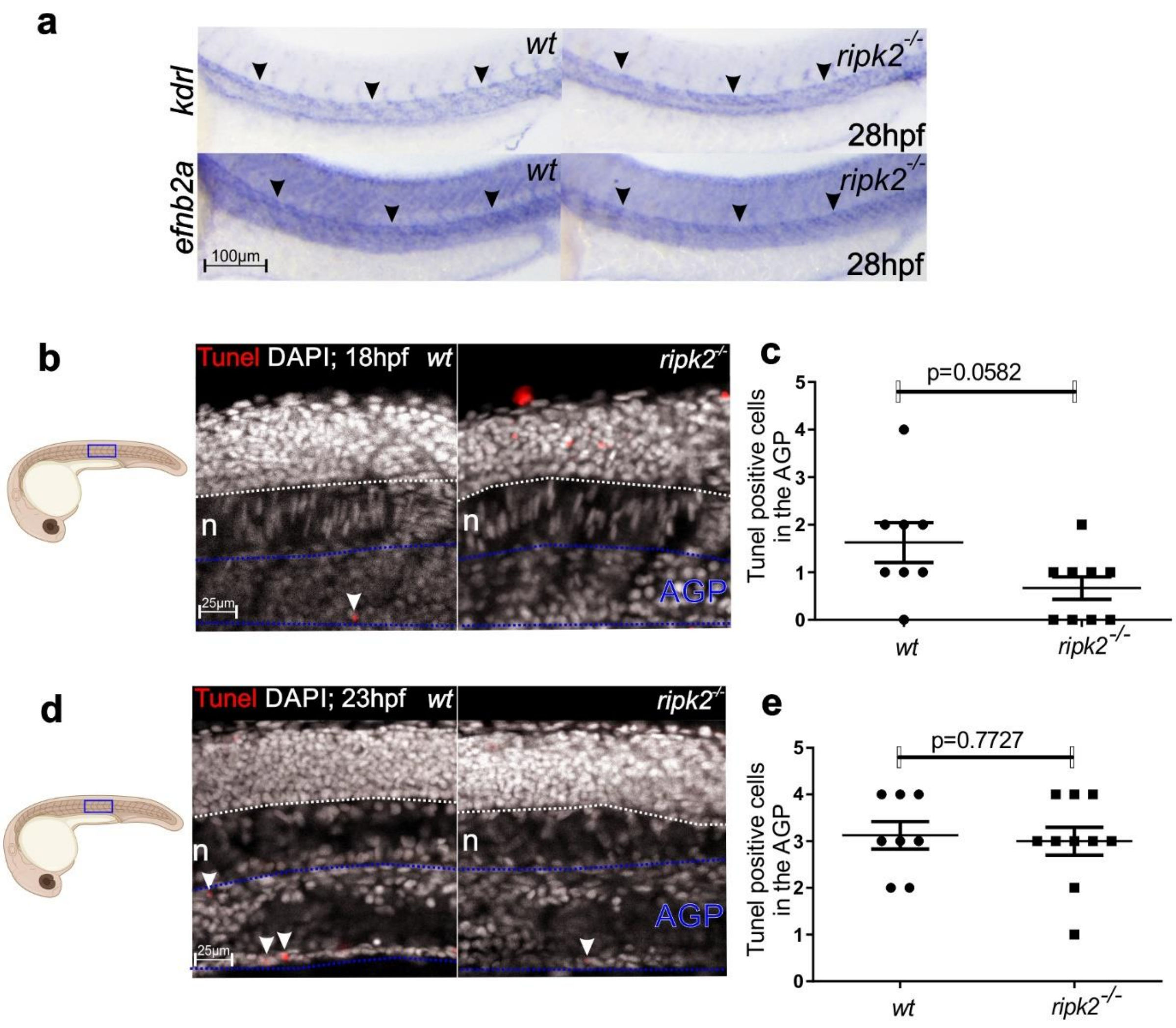

Supplementary Figure 3.

(a) Wildtype and *ripk2*$^{-/-}$ embryos were examined by WISH for *kdrl* and *efnb2a* expression in the trunk at 28hpf. Arrowheads denote *kdrl*$^{+}$ or *efnb2a*$^{+}$ expression. n=15 per condition. Images are representative of two independent experiments. **(b, d)** Single z-plane confocal images of the AGP region from 18 hpf (b) or 23 hpf (d) wildtype or *ripk2*$^{-/-}$ embryos assayed for TUNEL (red) and DAPI (white). Arrowheads denote apoptotic nuclei. All views are lateral, with anterior to the left. Illustrations created with BioRender.com. **(c, e)** Quantification of apoptotic nuclei from (b) and (d). Each dot represents total apoptotic cells in the AGP region per embryo. n=8, *wt*; n=9, *ripk2*$^{-/-}$ in (c). n=8 *wt*, n=10 *ripk2*$^{-/-}$ in (d). All quantifications are represented with mean ± SEM. Data was analyzed by unpaired two-tailed T-test (c,e). Source data are provided as a Source Data file.

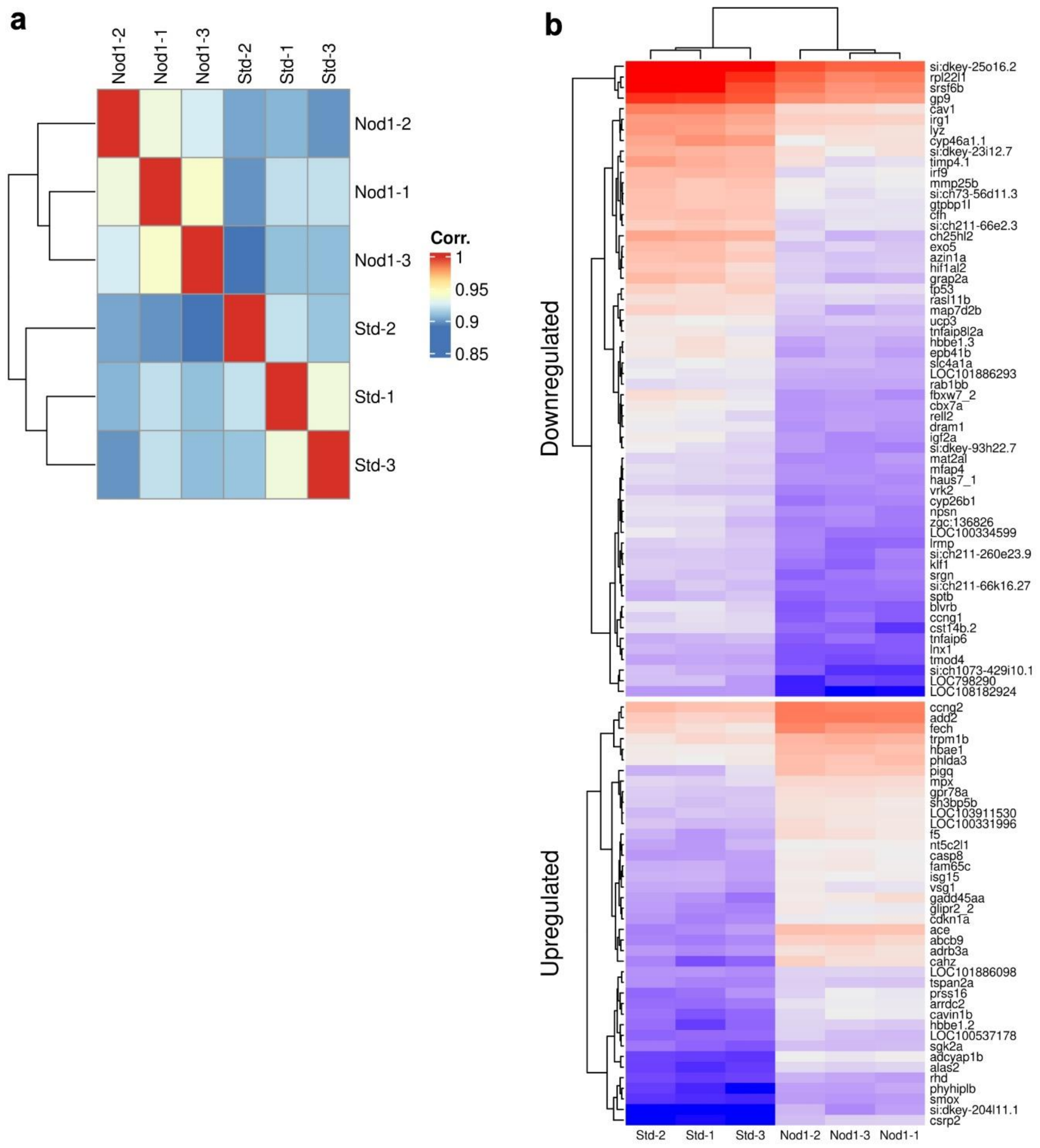

Supplementary Figure 4.

(a) *Tg*(*kdrl:mCherry)* embryos were injected with Std MO or Nod1 MO2 and *kdrl*$^{+}$ ECs were FACS purified at 22hpf for subsequent RNA-seq analysis. Sample correlation matrix displaying the overall similarity in expression profile for Std-control ECs and Nod1 deficient ECs. Red indicates stronger sample correlation. n=3 control; n=3 Nod1 MO2. **(b)** Heatmap of log_2 normalized counts in each sample of top 100 most significant genes with absolute log2 fold change >1 (ordered by p-value). Samples with relatively high expression of a given gene are marked in red and samples with relatively low expression are marked in blue. Lighter shades and white represent genes with intermediate expression levels. Samples and genes, after separating down- and up-regulated genes, have been ordered by hierarchical clustering with Euclidean distances and complete linkage.

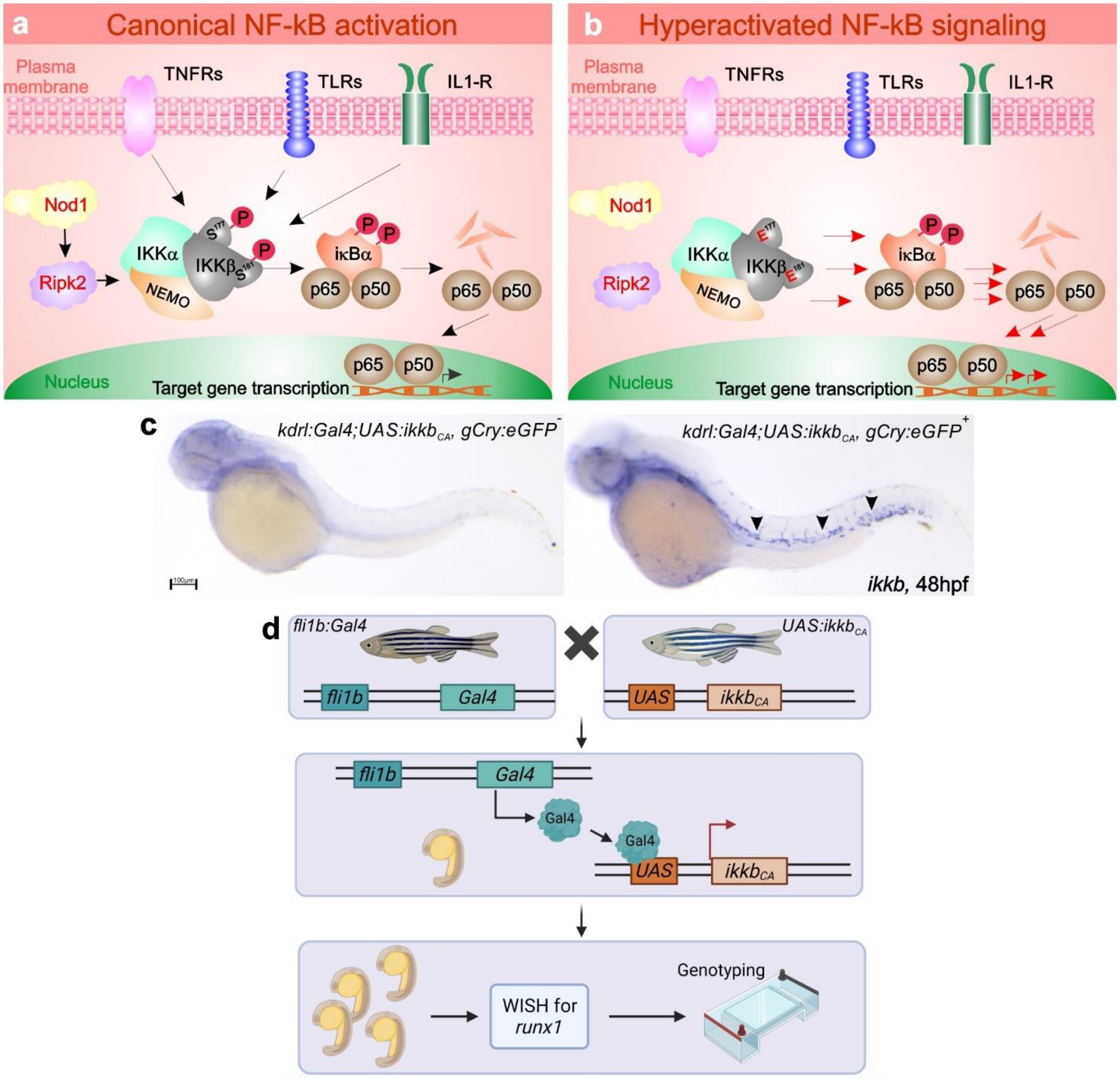

Supplementary Figure 5.

(a-b) Graphical illustration of canonical versus hyperactivated NF-kB signaling. Briefly, in the canonical NF-κB pathway (a), canonical NF-κB receptors TNFRs, IL-1R, TLRs, as well as Nod1 converge into IKKβ phosphorylation and subsequent activation. IKKβ phosphorylates IκBα, resulting in its detachment from the p65/p50 complex and degradation. Free p65/p50 heterodimers translocate to the nucleus and regulate gene transcription. In the hyperactivated NF-κB signaling pathway (b), overexpression of mutated IKKβ (IKKβ$^{S177E/S181E}$) renders this kinase constitutively active, resulting in the hyperactivation of NF-kB signaling in the absence of upstream canonical NF-kB activating signals. TLRs, toll-like receptors; TNFRs, tumor necrosis factor receptors; IL1-R, interleukin 1 receptor; IKKβ, inhibitor of nuclear factor kappa-B kinase subunit beta; IKKα, inhibitor of nuclear factor kappa-B kinase subunit alpha; Nod1, Nucleotide Binding Oligomerization Domain Containing 1; Ripk2, Receptor-interacting protein kinase 2. **(c)** Tg(*kdrl:Gal4; UAS:ikkb*$_{CA}$) embryos were examined by WISH for *ikkb* expression at 48hpf. Arrowheads denote *ikkb*$^{+}$ expression in the vasculature. n=5 per condition. All views are lateral, with anterior to the left. Images are representative of two independent experiments. **(d)** Schematic representation of the experimental design of Fig. 6h-i. Briefly, *Tg(fli1b:Gal4)* was crossed with *Tg(UAS:ikkb*$_{CA}$*)*. Offspring embryos were

interrogated by WISH for *runx1* at 26hpf and quantified blinded. *ikkb*$_{CA}$ was subsequently identified by genotyping, and embryos were grouped based on the absence or presence of the *ikkb*$_{CA}$ cassette. Illustration created with BioRender.com.

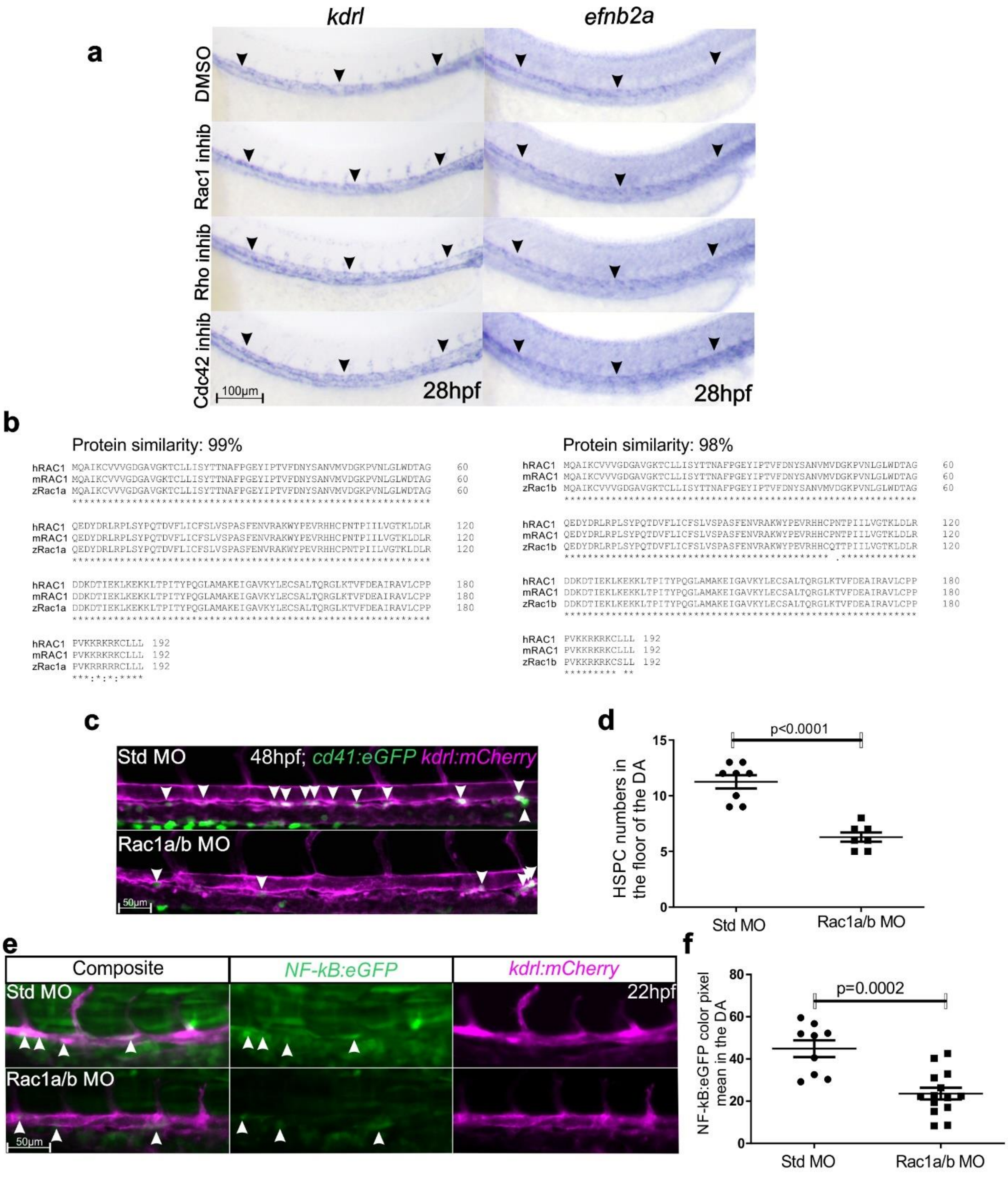

Supplementary Figure 6.

(a) DMSO and Small Rho GTPases inhibitor-treated embryos examined by WISH for *kdrl* and *efnb2a* expression in the trunk at 28hpf. Arrowheads denote *kdrl*$^{+}$ or *efnb2a*$^{+}$ expression in the dorsal aorta. n=15 per condition. Images are representative of two independent experiments. **(b)** Alignment of human (hRAC1, NP_008839.2) and mouse (mRac1, NP_033033.1) RAC1 with zebrafish Rac1a (zRac1a, NP_956065.1) and zebrafish Rac1b (zRac1b, NP_001034907.1) was performed using EM BL-EBI T-Coffee (https://www.ebi.ac.uk/Tools/msa/tcoffee/). **(c)** *cd41:eGFP; kdrl:mCherry* double-transgenic embryos

injected with Std MO or Rac1a/1b MOs and analyzed by live confocal microscopy at 48hpf. **(d)** Quantification of *cd41*$^+$*, kdrl*$^+$ HSPCs from (c). Each dot represents total HSPCs per embryo. n=8 Std MO, n=7 Rac1a/b MO. **(e)** Maximum projections of trunk regions of Std MO, or Rac1a/b MO-injected *kdrl:mCherry; NF-kB:eGFP* double-transgenic embryos visualized by confocal microscopy at 22 hpf. Arrowheads denote ECs with active NF-kB. All views are lateral, with anterior to the left. **(f)** Quantification of *NF-kB:eGFP* activity from (e). Each dot represents eGFP$^+$ mean pixel intensity within the *kdrl*$^+$ EC area delimited by two intersegmental vessels. n=9, Std MO; n=14, Rac1a/b MO. All quantifications are represented with mean ± SEM. Data was analyzed by unpaired two-tailed T-test (d, f). Source data are provided as a Source Data file.

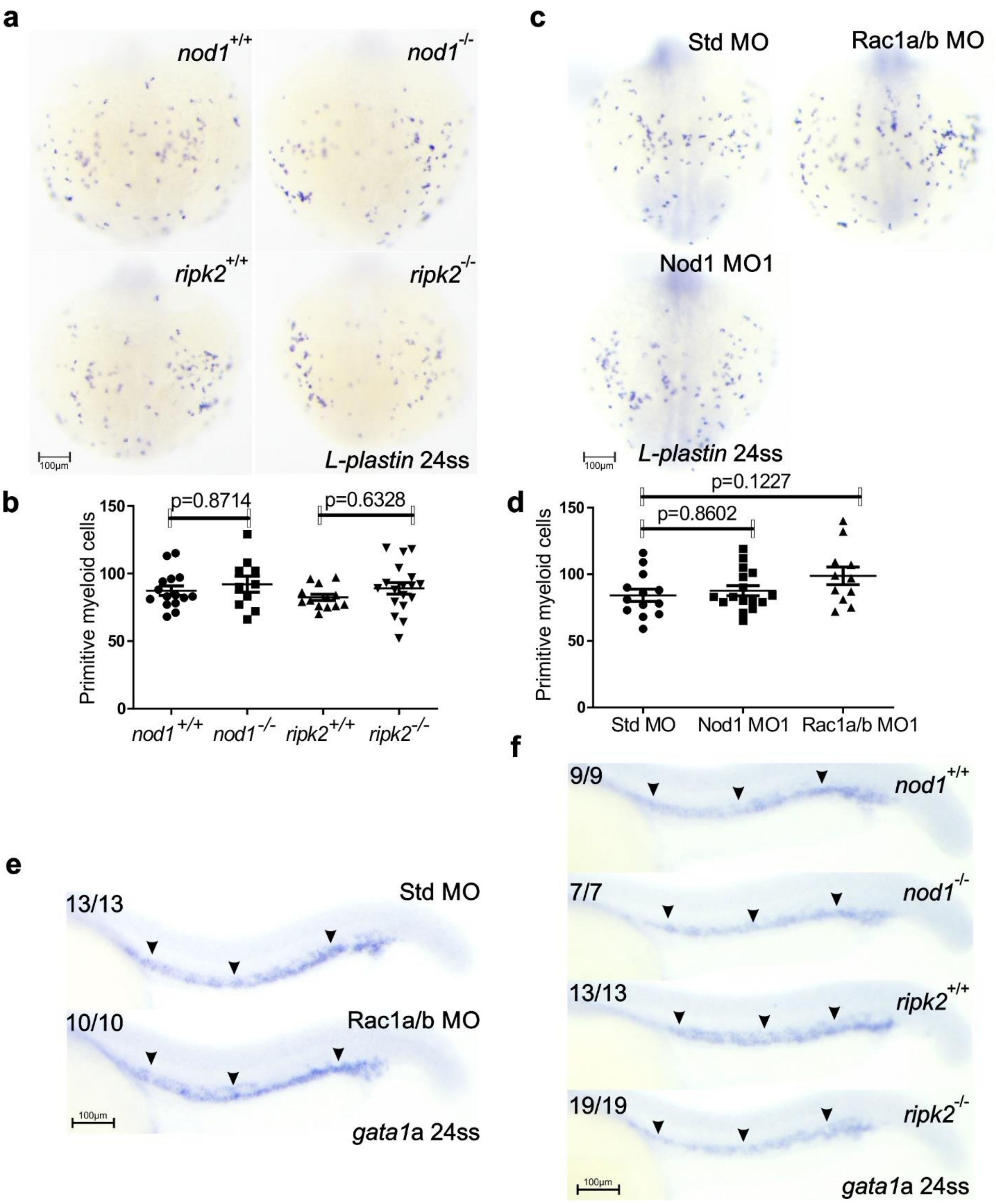

Supplementary Figure 7.

(a) *nod1*$^{+/+}$, *nod1*$^{-/-}$, *ripk2*$^{+/+}$ and *ripk2*$^{-/-}$ embryos were examined by WISH for *L-plastin* expression in the yolk ball at 24 somites (ss). **(b)** Quantification of *L-plastin*$^{+}$ myeloid cells from (a). Each dot represents total myeloid cells per embryo. n=14, 10, 13, 19 biological replicates from left to right. **(c)** Zebrafish embryos injected with Std MO, Rac1a/b MO and Nod1 MO1 were subjected to WISH for the myeloid cells marker *L-plastin* at 24ss. **(d)** Quantification of *L-plastin*$^{+}$ myeloid cells from (c). Each dot represents total myeloid cells

per embryo. n=13, 14, 11 biological replicates from left to right. **(e)** Zebrafish embryos injected with Std MO and Rac1a/b MO were subjected to WISH for erythrocytes marker *gata1a* at 24ss. Arrowheads denote *gata1a*$^{+}$ erythrocytes along the dorsal aorta. n=13 Std MO, n=10 Rac1a/b MO. **(f)** *nod1*$^{+/+}$, *nod1*$^{-/-}$, *ripk2*$^{+/+}$ and *ripk2*$^{-/-}$ embryos examined by WISH for *gata1a* expression at 24ss. Arrowheads denote *gata1a*$^{+}$ erythrocytes along the dorsal aorta. n=9 *nod1*$^{+/+}$, n=7 *nod1*$^{-/-}$, n=13 *ripk2*$^{+/+}$, n=19 *ripk2*$^{-/-}$. All views are lateral, with anterior to the left. All quantifications are represented with mean ± SEM. Data was analyzed by ordinary one-way ANOVA with Tukey's multiple comparisons test (b, d). Source data are provided as a Source Data file.

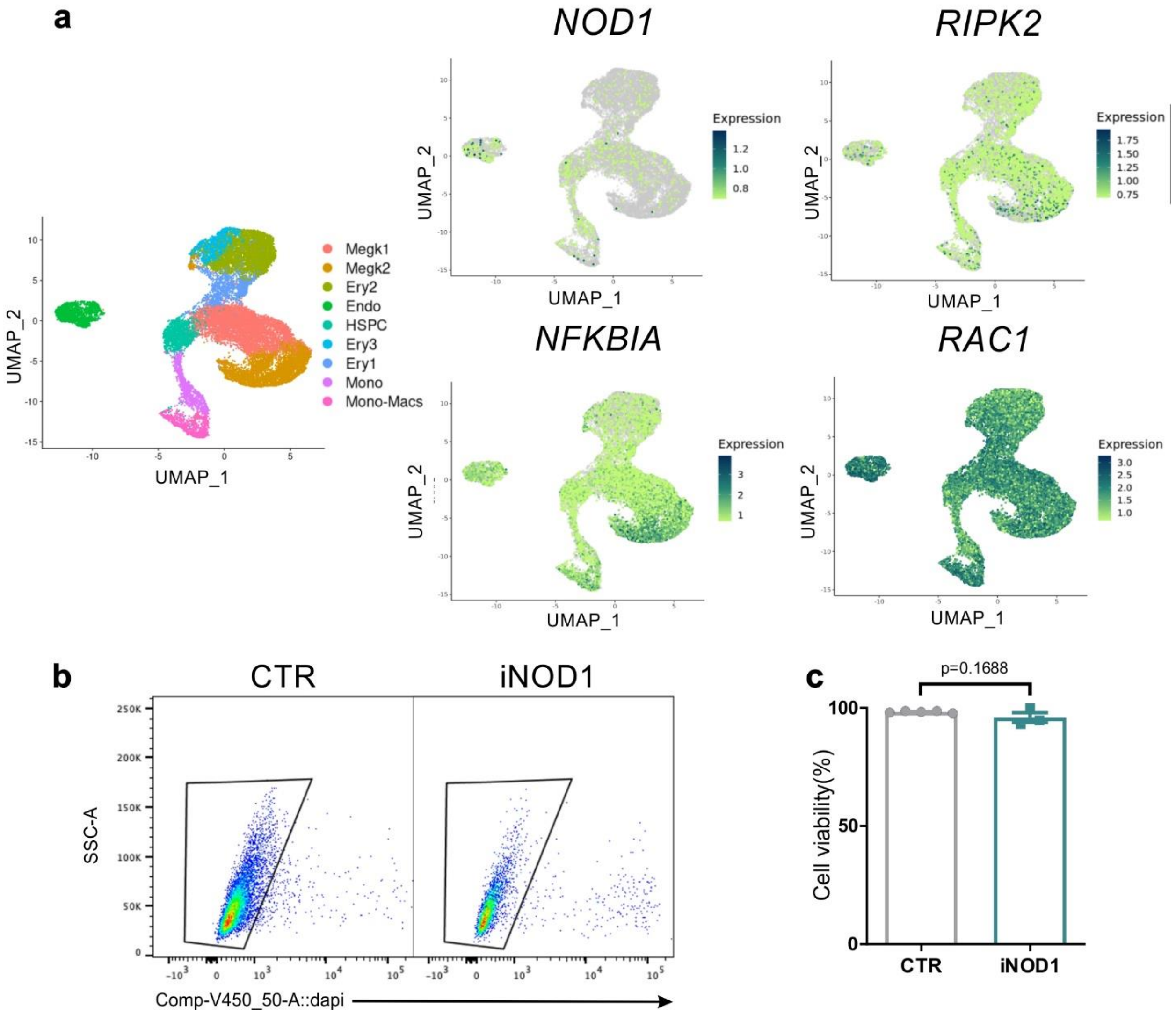

Supplementary Figure 8.

(a) UMAP visualization of the expression of indicated genes from scRNAseq dataset from definitive uncommitted hematopoietic progenitors and primed differentiated hematopoietic lineages. Each dot represents one cell. Grey denotes minimal expression, green intermediate, and blue high. MegK, Megakaryocyte progenitor; Ery, Erythroid progenitor; HSPC, Hematopoietic Stem and Progenitor cells; Mono, Monocytes progenitor; Mono-Macs, Monocytes - Macrophages progenitor. **(b-c)** Cell viability assessed by DAPI and flow cytometry in iNOD1 from day 2 of definitive hematopoietic differentiation. n=5, CTR; n=3, iNOD1. All quantifications are represented with mean ± SEM. Data was analyzed by unpaired two-tailed T-test (c). Source data are provided as a Source Data file.

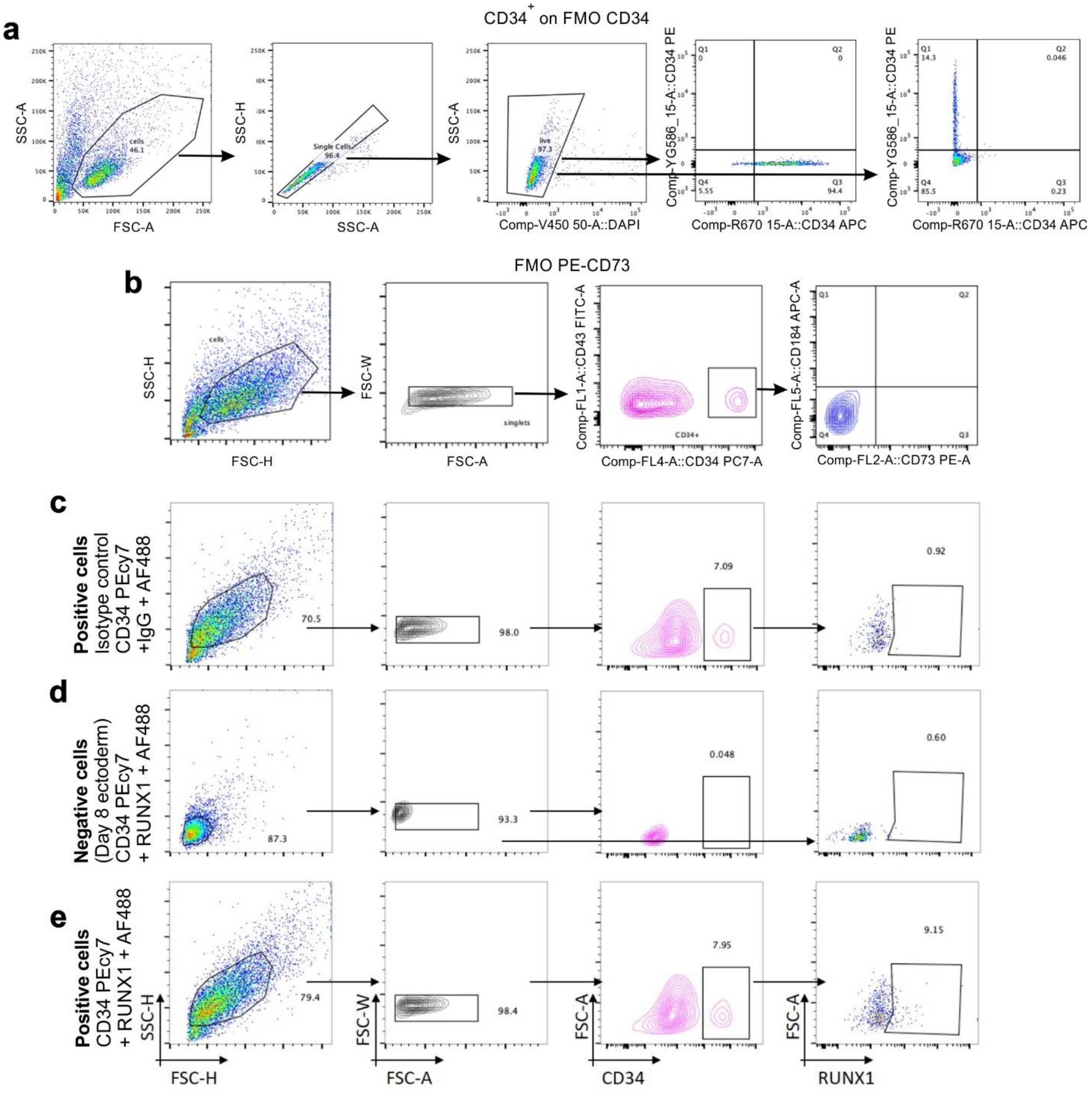

Supplementary Figure 9.

Representative gating strategies and RUNX1 antibody validation from flow cytometric dot plots in Fig. S8b (a), Fig. 8e (b), and Fig. 8f (c-e). **(a-e)** Live cells were gated on forward scatter (FSC-A or FSC-H) and side scatter (SSC-A or SSC-H), and singlets on side scatter (SSC-H, SSC-A) or forward scatter (FSC-A/FSC-W). The DAPI, CD73, 184 levels were measured within the CD34+ EC-like fraction. **(c-e)** RUNX1 antibody validation and gating strategy. Fixed and permeabilized cells were gated on forward scatter (FSC-H) and side scatter (SSC-H), and singlets on forward scatter (FSC-A/FSC-W). RUNX1 levels were measured within the CD34+ EC-like fraction. To establish staining specificity, two negative controls were used: positive cells (containing $CD34^+$ cells at day 9) stained with an isotype control and secondary antibody (c), and negative cells (d) from iPSC-derived ectoderm at day 8 (which do not contain mesoderm-derived cells neither RUNX1 expression) stained with CD34 and RUNX1 antibodies. (e) Positive cells (containing $CD34^+$ cells at day 9) were analyzed with CD34 and RUNX1 antibodies, resulting in ~9% of $RUNX1^+$ stained cells.

Table S1. Primer Sequences Used in This Study. Related to Experimental Procedures.

Gene	Accession	Directionality	Nucleotide sequence (5'->3')	Use
nod1	XM_002665060.6	F	GGCACGAACAATTTCGTTTT	gRNA validation
		R	CTGTTTTAGTGCTGCTGCGC	
		F	GCGGTATTGAGGTTCTGGCT	qPCR
		R	TGTGGTTTTGGTAAAGGCCCA	
		F	GTGAAGGTGTTGGGGTGAGT	sa17969 mutant line validation
		R	CAAACAAGTGACCACCATGC	
		F	GATCTTTCATTTCATTTTTCAGAGCCCGA	Morpholino validation
		R	GATCTCGGGCTCTGAAAAATGAAATGAAA	
ripk2	NM_194411.2	F	GGGTCTGCCGTCATCATTAAT	*ripk2$^{z40/z40}$* mutant line validation
		R	GTGAGGGGTTGTATGGCAAGA	
		F	GGGTCTAGTACGTAGGCTGGA	qPCR
		R	CACCGGTAATGTGCTGGTGA	
rac1a	NM_199771.1	F	CCGCTCTTGTTTTGCGTGTT	qPCR
		R	TTTTTCCCACAGCCCCGTCC	
		F	ACTCATGGATATCGGCAAGC	gRNA validation
		R	CGGTCGAAGCCTGTCATAAT	
rac1b	NM_001039818.1	F	GGGGTTTTTCATCAGTTCCGC	qPCR
		R	TTTTACCCACAGCCCCGTCA	
		F	AGGAAGCTGCCATGGTGTTA	gRNA validation
		R	CATGTCTGCAGGTTTGTGCT	
nod2	NM_001328044.1	F	ACACA CC CACAACAGGTTC	qPCR
		R	GAAGAGGGACTGCGATGCAA	
cdc42	NM_001018120.2	F	CTCTGACGCAGCGAGGTC	qPCR
		R	TGCGTTTCAGGAGGTTCGAG	
rho	NM_131084.1	F	CCGGAGCCCATACGAATACC	qPCR
		R	AGGAAGAACATGTAGGCCGC	
ef1a	NM_131263.1	F	GAGAAGTTCGAGAAGGAAGC	qPCR
		R	CGTAGTATTTGCTGGTCTCG	
ciita	XM_005163915.4	F	GCACTGTGGTTCAGACAGGA	qPCR
		R	CAACCGTACCATCAGCAGGT	
nlrc5	NM_001386270.1	F	TCCTTCCTGTCATGTTGTCTC	qPCR
		R	TCAGCTTGGTGCCTGAGTTC	
nlrx1	XM_680389.9	F	TCCACACAGTGCATCTGTACC	qPCR
		R	CAGAGATGTCCGAACCCTCG	
P2A	N/A	F	GAATTCACCCGGGTACCGCTACTAACTTCAGCCTGCTGAAG	*P2A* cloning
		R	GCCATTTCTACCGGTACAGGTCCAGGGTTCTCCTCC	
$ikkb_{CA}$	N/A	F	CGGCAAGGGTCGACTCTAGAATGAGCCGTCCGCCC	*$ikkb_{CA}$* cloning
		R	GCTGAAGTTAGTAGCGGTACCAGCCAGATCCTGTCCTCC	
$ikkb_{CA}$– mRFP	N/A	F	GAACCGCCTGCAGAGTTTAG	*Fli1b:Gal4;UAS:$ikkb_{CA}$* genotyping
		R	GTCCTCGAAGTTCATCACGC	
Rac1-Q61R	N/A	F	CGGATATCGATAAGCTTGATATCGAATTCATGCAGGCCATCAAGTGTG	rac1-Q61R cloning
		R	CCGTATCGATACTAGTCCTGCAGGTTACAACAGCAGGCATTTTCTCTTCC	

Cre-cmlc2-eGFP	N/A	F	TTGCGCTGATGCCCAGTTGCGGCC GCGAGGATCATAATCAGCCATAC	*cmlc2-eGFP* cloning
		R	CGAATTCCTGCAGCCCGGGGCCTG CAGGACTAGTATCGATACGGAC	
ikkb	N/A	F	ATATGAATTCatgagccgtccgccctccatgc agcca	Ikkb cloning
		R	CCTATCTCGAGtcaagccagatcctgtcctc cgttca	

Table S2. Morpholinos (MOs) Used in This Study. Related to Experimental Procedures.

Name	Sequence (5'-3')	Concentration	Reference
Std MO	CCTCTTACCTCAGTTACAATTTATA	0.4mM	Gene Tools
Nod1 MO1	TTTCATTTCATTTTTCAGAGCCCGA	0.8mM	This study
Nod1 MO2	ACCAAATAAACATTACCTGGTCTGT	1mM	(Oehlers et al., 2011)
Ripk2 MO	GCTCCATGTTTCTGGACATTAGGAG	1mM	This study
Rac1a MO	CCACACACTTTATGGCCTGCATCTG	0.28mM	(Mikdache et al., 2020)
Rac1b MO	CCACACACTTGATGGCCTGCATGAC	0.28mM	(Epting et al., 2015)

Table S3. Chemicals Used in This Study. Related to Experimental Procedures.

Name	Use	Concentration used	Reference
C12-iE-DAP	Nod1 agonist	250ng/µl – 400ng/µl	InvivoGen (tlrl-c12dap)
Rho Kinase Inhibitor III	Rho inhibitor	15 µM	(Weiser et al., 2009)
Hydrochloride	Rac1 inhibitor	50 µM	(Nussbaum et al., 2013)
ML141	Cdc42 inhibitor	40 µM	(Stanganello et al., 2015)
DAPI	Nuclei staining	1:1000	N/A
Nodinitib-1 (iNOD1)	Nod1 inhibitor	15µM-30µM	(Correa et al., 2011)

Table S4. Antigens and Secondary Antibody Used in This Study. Related to Experimental Procedures.

Antigen	Clone name	Fluorophore	Manufacturer	Catalog number	Lot number	Dilution
CD34	4H11	PE	Thermo Fisher	12034942	N/A	1:200
CD43	eBio84-3C1	APC	ebioscience	17043942	N/A	1:100
CD43	MEM-59	FITC	Biolegend	**315204**	B293675	1:100
CD34	4H11	FE-Cy7	Invitrogen	25-0349-42	2011157	1:100
CD73	AD2	PE	BD	550257	5020998	1:25
CD184	12G5	APC	BD	555976	6154735	1:100
RUNX1	D33G6	N/A	CST	4336	5	1:500
Rabbit IgG	DA1E	N/A	CST	3900	49	1:2500
**Secondary Ab**	**Clone name**	**Fluorophore**	**Manufacturer**	**Catalog number**	**Lot number**	**Dilution**
Anti-Digoxigenin-AP	N/A	N/A	Roche	11093274910	57696520	1:5000
Strepavidin	N/A	Alexa 647	Thermo Fisher	S21374	2145944	1:500
Goat anti Rabbit	N/A	AF647	Invitrogen	A21245	1863958	1:1000
