# Supplementary material for: Nod1-dependent NF-kB activation initiates hematopoietic stem cell specification in response to small Rho GTPases
Source: Nat Commun. 2023 Nov 23;14:7668. doi: 10.1038/s41467-023-43349-1 (PMC10667254; doi:10.1038/s41467-023-43349-1)
Supplement: Supplementary file 3 — Reporting Summary [file 41467_2023_43349_MOESM3_ESM.pdf]

nature portfolio

Corresponding author(s): Raquel Espin-Palazon

Last updated by author(s): Nov 1, 2023

Reporting Summary

Nature Portfolio wishes to improve the reproducibility of the work that we publish. This form provides structure for consistency and transparency in reporting. For further information on Nature Portfolio policies, see our Editorial Policies and the Editorial Policy Checklist.

Statistics

For all statistical analyses, confirm that the following items are present in the figure legend, table legend, main text, or Methods section.

n/a	Confirmed	
☐	☒	The exact sample size (*n*) for each experimental group/condition, given as a discrete number and unit of measurement
☐	☒	A statement on whether measurements were taken from distinct samples or whether the same sample was measured repeatedly
☐	☒	The statistical test(s) used AND whether they are one- or two-sided<br>*Only common tests should be described solely by name; describe more complex techniques in the Methods section.*
☒	☐	A description of all covariates tested
☒	☐	A description of any assumptions or corrections, such as tests of normality and adjustment for multiple comparisons
☐	☒	A full description of the statistical parameters including central tendency (e.g. means) or other basic estimates (e.g. regression coefficient) AND variation (e.g. standard deviation) or associated estimates of uncertainty (e.g. confidence intervals)
☐	☒	For null hypothesis testing, the test statistic (e.g. *F*, *t*, *r*) with confidence intervals, effect sizes, degrees of freedom and *P* value noted<br>*Give P values as exact values whenever suitable.*
☒	☐	For Bayesian analysis, information on the choice of priors and Markov chain Monte Carlo settings
☒	☐	For hierarchical and complex designs, identification of the appropriate level for tests and full reporting of outcomes
☒	☐	Estimates of effect sizes (e.g. Cohen's *d*, Pearson's *r*), indicating how they were calculated

Our web collection on statistics for biologists contains articles on many of the points above.

Software and code

Policy information about availability of computer code

Data collection	Flow cytometry data were collected using FACSChorus 2.0, version 1.1.20.0 (BD).<br>Confocal images were acquired with Zen Black, version 14.0.27.201.<br>Microscopic fluorescent images were acquired with Leica Application Suite X, version 3.7.0.20979.<br>Bright-field images were acquired with Leica Application Suite X, version 3.7.0.20979.<br>Quantitative PCR data were collected using Bio-Rad CFX Maestro 2.0, version 5.0.021.0616
Data analysis	Microscopic images were processed with ImageJ 1.53f51.<br>Flow cytometric data were analyzed using FlowJo 10.3 or 10.8.1 software.<br>Graphpad Prism 5, 9, or 10 were utilized to perform statistical analysis and represent data.<br>All figures were assembled in CorelDraw Graphics Suite X6 and 2020.<br>Protein alignments were calculated using EM BL-EBI T-coffee (https://www.ebi.ac.uk/Tools/msa/clustalo/)<br>RNA-seq data: For differential expression analysis, we used Salmon v1.9.0 (PMID: 28263959) to align trimmed reads to the GRCz11 (RefSeq assembly GCF_000002035.6) RNA sequences, annotation release 106. Then, we used DESeq v1.40.1 to detect differential expression.<br>R package v3.17.0 was used to compute differentially expressed genes. For GO enrichment, we used the Coriell convenience functions to detect if significantly down-regulated genes (adjusted p-value <0.05) were overrepresented in any biological process using Fisher's exact test with false discovery rate (FDR) controlled at <0.05 by the Benjamini-Hochberg method. For the biological processes of "immune system process", "embryonic hemopoiesis", "erythrocyte differentiation", and "myeloid differentiation", we identified genes annotated to these processes, using the R package v3.17.0, which also had adjusted p-values <0.05 and decreased in expression by at least one half (log_2-fold change below -1). Complex Heatmap was used to plot the log_2 expression of these selected genes.<br>For the human iPSCs derived dataset, gene expression values were obtained using the web portal at https://lab.antonellafidanza.com/, the website https://singlecell.mcdb.ucla.edu/Human-HSC-Ontogeny/, or NCBI's Gene Expression Omnibus (GEO) database (Zeng et al., 2019)

under accession number GSE136202 [https://www.ncbi.nlm.nih.gov/geo/query/acc.cgi?acc=GSE136202]. For the in vivo dataset, data were analyzed using Seurat R package (Stuart et al., 2019) and Flowio software (Tree Star, OR, USA)

For manuscripts utilizing custom algorithms or software that are central to the research but not yet described in published literature, software must be made available to editors and reviewers. We strongly encourage code deposition in a community repository (e.g. GitHub). See the Nature Portfolio guidelines for submitting code & software for further information.

Data

Policy information about availability of data

All manuscripts must include a data availability statement. This statement should provide the following information, where applicable:

- Accession codes, unique identifiers, or web links for publicly available datasets
- A description of any restrictions on data availability
- For clinical datasets or third party data, please ensure that the statement adheres to our policy

The raw RNA-seq data generated in this study have been deposited in the NCBI GEO database with accession number GSE224708 [https://www.ncbi.nlm.nih.gov/geo/query/acc.cgi?acc=GSE224708]. All data generated in this study are available in Article and Supplementary Information. Source data are provided with this paper.

Research involving human participants, their data, or biological material

Policy information about studies with human participants or human data. See also policy information about sex, gender (identity/presentation), and sexual orientation and race, ethnicity and racism.

Reporting on sex and gender	N/A
Reporting on race, ethnicity, or other socially relevant groupings	N/A
Population characteristics	N/A
Recruitment	N/A
Ethics oversight	N/A

Note that full information on the approval of the study protocol must also be provided in the manuscript.

Field-specific reporting

Please select the one below that is the best fit for your research. If you are not sure, read the appropriate sections before making your selection.

☒ Life sciences ☐ Behavioural & social sciences ☐ Ecological, evolutionary & environmental sciences

For a reference copy of the document with all sections, see nature.com/documents/nr-reporting-summary-flat.pdf

Life sciences study design

All studies must disclose on these points even when the disclosure is negative.

Sample size	Based on our previous observations and preliminary data, 10-15 animals per group for WISH and qPCR experiments, and 4-10 animals for confocal live imaging are sufficient for 95% statistical power.
Data exclusions	Whole-mount in situ hybridization for runxl and cmyb in zebrafish embryos to address cell count were only excluded in those individuals whose staining failed.
Replication	Each experiment was performed at least three independent times to ensure reproducibility. Alternatively, at least two different methods or assays were used to confirm a particular result. For example, to confirm the loss of hematopoietic stem cells in loss of function experiments, two or more strategies were used for each particular gene, including one or two gene specific morpholinos, null mutant, CRISPANT, and chemical inhibition. Hematopoietic stem cell numbers were assessed when possible, by two different methodologies, including in situ hybridization for runxl or cmyb, and live confocal analysis of kdrl:mCherry; cd41:eGFP double transgenic embryos. For RNA-seq, three independent biological replicates were collected and analyzed.
Randomization	Zebrafish offsprings were pooled randomly into control and experimental groups for injection-based experiments. In those experiments involving mutant zebrafish, randomization was not possible, but single-blind experimental analysis was ensured with methods described below.
Blinding	Experimental analysis were performed blinded for zebrafish genetic experiments. In some cases, the embryos were genotyped after the experimental procedure was performed. In other cases, control and experimental conditions were labeled with numbers by other lab mates

and revealed once quantification is complete, to ensure single-blind study.

Reporting for specific materials, systems and methods

We require information from authors about some types of materials, experimental systems and methods used in many studies. Here, indicate whether each material, system or method listed is relevant to your study. If you are not sure if a list item applies to your research, read the appropriate section before selecting a response.

Materials & experimental systems

n/a	Involved in the study
☐	☒ Antibodies
☐	☒ Eukaryotic cell lines
☒	☐ Palaeontology and archaeology
☐	☒ Animals and other organisms
☒	☐ Clinical data
☒	☐ Dual use research of concern
☒	☐ Plants

Methods

n/a	Involved in the study
☒	☐ ChIP-seq
☐	☒ Flow cytometry
☒	☐ MRI-based neuroimaging

Antibodies

Antibodies used

Antigen	Clone name	Fluorophore	Manufacturer	Catalog number	Lot number	Dilution
CD34	4H11	PE	Thermo Fisher	12034942	N/A	1:200
CD43	eBio84-3C1	APC	ebioscience	17043942	N/A	1:100
CD43	MEM-59	FITC	Biolegend	315204	B293675	1:100
CD34	4H11	FE-Cy7	Invitrogen	25-0349-42	2011157	1:100
CD73	AD2	PE	BD	550257	5020998	1:25
CD184	12G5	APC	BD	555976	6154735	1:100
RUNX1	D33G6	N/A	CST	4336	5	1:500
Rabbit IgG	DA1E	N/A	CST	3900	49	1:2500

Secondary Ab	Clone name	Fluorophore	Manufacturer	Catalog number	Lot number	Dilution
Anti-Digoxigenin-AP	N/A	N/A	Roche	11093274910	57696520	1:5000
Strepavidin	N/A	Alexa 647	Thermo Fisher	S21374	2145944	1:500
Goat anti Rabbit	N/A	AF647	Invitrogen	A21245	1863958	1:1000

Validation

The antibodies were validated as indicated in the main manuscript/supplementary information.

Eukaryotic cell lines

Policy information about cell lines and Sex and Gender in Research

Cell line source(s)

Human induced pluripotent stem cell (hiPSC) line —SFCi55 — was produced by Roslin Cells (Edinburgh), by reprogramming fibroblasts derived from skin cells of a female individual with O Rhesus negative blood type (R Biomedical LTD, Edinburgh UK under REC 1/AL/0020 ethical approval).

Authentication

N/A

Mycoplasma contamination

Cell lines were routinely tested for mycoplasma by PCR

Commonly misidentified lines
(See ICLAC register)

N/A

Animals and other research organisms

Policy information about studies involving animals; ARRIVE guidelines recommended for reporting animal research, and Sex and Gender in Research

Laboratory animals

nod1 mutant zebrafish (Danio rerio) strain (sa17969) was obtained from the Zebrafish International Resource Center (ZIRC). ripk2z40 zebrafish mutants were kindly donated by Michael Jurynec36. Other zebrafish lines used in this study were: wt AB* (ZIRC), Tg(cmyb:eGFP)zf169 17, Tg(kdrl:HsHRAS-mCherry)s896 12(referred to as kdrl:mCherry throughout the manuscript), Tg(-6.0itga2b:eGFP)la2 29 (referred to as cd41:eGFP throughout manuscript), Tg(NF-kB:eGFP)nc1 30, Tg(Rag2:eGFP)zdf8 71, Tg(kdrl:Gal4) sd14 72, Tg(γcry:eGFP, 14xUAS:ikkbCA-P2A-mRFP)is501 referred to as Tg(UAS:ikkbCA) throughout manuscript (generated in this work), fli1btpl50Gt 44, referred as Tg(fli1b:Gal4) throughout the manuscript, and various intercrosses of these lines were utilized. Above zebrafish embryos and adults were mated between 3-15 months of age, staged, raised, and processed as described (Westerfield, 2000) in a circulating aquarium system at 28°C. All offsrping ages are noted in each figure.

Wild animals	N/A
Reporting on sex	All experiments in this study were conducted on zebrafish animals at the embryo or larvae stages. At these stages, sex cannot be determined, since in this specie, sex is not determined genetically.
Field-collected samples	N/A
Ethics oversight	The zebrafish research in this study was performed according to the Guidelines for Ethical Conduct in the Care and Use of Animals. All experiments in zebrafish were performed according to Iowa State University Animal Care and Use Committee IACUC-20-025 and IACUC-20-024 approved protocols, and in compliance with ARRIVE guidelines, and the American Veterinary Medical Association (2020) and NIH guidelines for the humane use of animals in research.

Note that full information on the approval of the study protocol must also be provided in the manuscript.

Plants

Seed stocks	N/A
Novel plant genotypes	N/A
Authentication	N/A

Flow Cytometry

Plots

Confirm that:

☒ The axis labels state the marker and fluorochrome used (e.g. CD4-FITC).

☒ The axis scales are clearly visible. Include numbers along axes only for bottom left plot of group (a 'group' is an analysis of identical markers).

☒ All plots are contour plots with outliers or pseudocolor plots.

☒ A numerical value for number of cells or percentage (with statistics) is provided.

Methodology

Sample preparation	For qPCR or RNA seq, Tg(kdrl:mCherry) zebrafish embryos were dissociated as followed (Barakat, Campbell, Espin-Palazon, Zebrafish 2022):<br>1. Remove embryos from their chorion.<br>2. Transfer up to 50 zebrafish embryos to 1.5 ml tube. Wait 5-10 seconds until embryos deposit to the bottom by gravity.<br>3. Remove E2 embryo media. Avoid touching or damaging the embryos.<br>4. Add 500 µl per tube of PBS with Ca2+ Mg2+. Wait 5-10 seconds until embryos deposit to the bottom.<br>5. Remove PBS with Ca2+ Mg2+.<br>6. Add 500 µl per tube of Liberase TM (Roche, cat. no. 05401119001) at working solution (50 µg/ml in PBS with Ca2+ Mg2+).<br>7. Gently perform an initial mechanical dissociation with a P1000 pipette set up to 400 µl (to avoid the introduction of bubbles) by gently pipetting up and down 5-10 times.<br>8. Incubate the tubes in a rotator at 28.52C 5 min for 22 hours post-fertilization (hpf) embryos.<br>9. Pipette the embryos gently up and down with a P1000 set up to 400 µl until the solution is homogeneous.<br>10. Transfer the solution to a 1ml syringe attached to a 20 gauge needle (Fisher Scientific; 148265C) containing a 30 µm nylon mesh (Fisher Scientific; NC9084441) between the syringe and the needle. Gentle push the plunger to filter the solution. Deposit the filtered solution into a flow cytometry tube.<br>11. Fill up the flow cytometry tube with 3 ml of cold PBS without Ca2+ Mg2+ (or cold FACS buffer, see below, for FACS applications).<br>12. Centrifuge the tubes at 300 rcf for 7 minutes at 4°C.<br>13. Discard the supernatant by gently removing the liquid with a P1000 pipette. Keep the cells on ice from this point. Leave 150-200 µl of the solution at the bottom of the tube containing the cells. Discarding the supernatant by other methods such as decanting will considerably reduce the number of cells collected.<br>14. Resuspend the cells by gently pipetting the cells with a P1000. Avoid the introduction of air.
Instrument	BD FACS Melody Cell Sorter
Software	BD FACS Chorus 2.0 Version 1.1.20.0
Cell population abundance	kdrl+ cells represented 2-5% from parent. Aminimum of 200,000 cells per sample were analyzed.

Gating strategy

All gating strategies are provided in the Supplementary information - supplementary figure 9.
For Tg(kdrl:mCherry) zebrafish embryos. Gates were set to unstained cells with no primary antibody present.
1. Gate your cell population using FSC-A and SSC-A
2. Eliminate doublets:
a. Y axis: FSC-W. X axis: FSC-A. Gate all the events that are grouped together. Double click inside of the gate.
b. Y axis: SSC-W. X axis: SSC-A. Gate all the events that are grouped together. Double click inside of the gate.
3. Eliminate dead cells using SytoxRed: Y axis: SSC-A. X axis: Comp APC-A. Gate all the events that are grouped together.
4. Asses fluorescence by using the mCherry (red) channel.

☒ Tick this box to confirm that a figure exemplifying the gating strategy is provided in the Supplementary Information.
